# From Blocks to Bots: The STEM Potential of Technology-Enhanced Toys in Early Childhood Education

**DOI:** 10.3390/bs16010161

**Published:** 2026-01-22

**Authors:** Dimitra Bourha, Maria Hatzigianni, Trifaini Sidiropoulou, Michael Vitoulis

**Affiliations:** 1 Department of Early Childhood Education & Care, University of West Attica, 122 43 Egaleo, Greece; mhatzigianni@uniwa.gr (M.H.); sidirofg@uniwa.gr (T.S.); 2 Department of Early Childhood Education & Care, International Hellenic University, 574 00 Thessaloniki, Greece; vitoulis@ihu.gr

**Keywords:** early childhood, STEM, digital technology, technology-enhanced toys, free play

## Abstract

Incorporating STEM (Science, Technology, Engineering, and Mathematics) into early childhood education has been associated with children’s holistic development. STEM education not only enhances critical thinking, creativity, problem-solving, and other 21st-century skills but also contributes significantly to cognitive growth, emotional regulation, and social abilities. Within the early childhood context, the use of play and toys emerges as a natural and powerful medium for introducing STEM concepts in developmentally appropriate and engaging ways. Play and toys have a prominent role, and previous studies have provided strong evidence on their educational benefits. Toys enhanced with technological characteristics (Technology-Enhanced Toys—TETs), such as coding and interactive toys, are increasingly being viewed as cultural tools that mediate learning and nurture cognitive and collaborative skills among young learners. However, the impact TETs have on young children’s STEM learning remains largely unexplored. This qualitative observational study, grounded in a socio-cultural perspective, explored how 37 children aged 3 to 4 years in four early childhood settings in Greece exhibited STEM-related behaviours during free play with technology-enhanced toys. Data were collected through systematic video recordings and written observations over a three-month period that involved interacting with various TETs, such as Bee-Bot, Coko Robot, a remote-controlled dog, and others. Results indicate that playing with TETs enhanced problem-solving, computational thinking, and collaboration, thus affirming the positive influence of digital technology and the potential of TETs to enrich early STEM education. Implications for equity, the importance of teachers’ professional development in effectively integrating TETs into early childhood curricula and the need for further research will also be discussed.

## 1. Introduction

Science, Technology, Engineering, and Mathematics (STEM) education has begun to play a central role in early childhood settings, with increasing efforts to embed STEM in more playful, conceptual, integrated learning, tangible experiences, and inquiry-based approaches that intentionally build on young children’s learning needs (Brenneman et al., 2019; Johnston et al., 2022; H. Li et al., 2021). Evidence shows that participating in STEM-related activities in early years supports the development of fundamental skills, including collaboration, problem-solving, and critical thinking, which are vital 21st-century competencies (Nikolopoulou, 2023; Stone, 2024). Longitudinal studies also indicate that early exposure to STEM concepts enhances children’s creative thinking, teamwork, and interest in science and technology throughout their educational journey (Linder & Eckhoff, 2020; Veziroglu-Celik et al., 2025). Furthermore, such opportunities foster positive attitudes, motivation for scientific inquiry, and cognitive flexibility (Arohunmolase, 2025; Bagiati & Evangelou, 2015).

Early childhood education and care (ECEC) educators promote STEM learning by encouraging children’s play-based activities. Providing child-initiated exploration opportunities with a variety of materials and facilitating peer interactions allows children to be active, spontaneous, and self-directed (Edwards, 2017). Free play enhances children’s agency, enabling them to pursue their interests, make independent decisions, negotiate social roles, and explore their unique ideas (Arnott, 2018). Extensive research links free play to the development of language and social-emotional skills, executive functions, creativity, self-regulation, cognitive flexibility, resilience, and emotional intelligence (Colliver et al., 2022; Madanipour & Cohrssen, 2020; Veiga et al., 2017). However, not all children benefit equally, as socio-economic factors often influence access to enriching play opportunities, highlighting the importance of education and policy in ensuring equitable access for all children (Cankaya et al., 2023).

New forms of play have emerged in our digital era. For instance, technology-enhanced toys (TETs), including programmable robots, electronic construction kits, and interactive multimedia devices, are widely recognised as “possibility tools” in early learning contexts. TETs offer additional opportunities for young children to engage in meaningful, self-directed exploration of STEM ideas, fostering the development of engineering habits of mind (EHoM), creative problem-solving, computational thinking, and early scientific inquiry (Hu et al., 2024; Kewalramani et al., 2020). The dynamic and flexible nature of open-ended play with TETs allows children to explore, experiment, collaborate, and build knowledge through imaginative and active learning. However, the educational value of TETs relies on proper access, supportive family environments, and effective pedagogical practices by teachers and educators (Bourha et al., 2024; S. Li, 2021; Madanipour & Cohrssen, 2020; Park et al., 2017; Wan et al., 2021).

Despite interest in STEM learning using play-based technologies, the empirical literature is dominated by samples of 5 year olds and older, often treating the early childhood years as a developmentally uniform category. Consequently, child-directed play moments with TETs in early childhood education and care (ECEC) settings with groups of children aged 3–4 years remain underexamined. Existing research predominantly emphasises individual tasks or adult-directed activities, offering limited insight into the progression of play-based pedagogy for STEM learning as it manifests in children’s social interactions and addresses their real-life needs in early childhood settings (Sikder, 2024). Findings from studies involving older preschool and early primary-aged children cannot be accurately applied to younger cohorts, as there are distinct differences in cognitive flexibility, symbolic representation, executive functioning, and peer collaboration due to developmental changes during the critical 3 to 4-year period (Wolak & Kim, 2023). This highlights the necessity for age-specific inquiry. The present study examines free play among 3- to 4-year-old children with TETs, investigating how it can facilitate STEM-related learning in a population that engages cognitively and socially in ways fundamentally different from those of older children (Kewalramani et al., 2020).

The research is therefore grounded in both sociocultural theory (viewing play as mediated action) and the concept of developing habits of mind through engineering; these two ideas together provide a useful theoretical framework to understand how young children’s behaviours related to STEM emerge through use of TETs and their interactions with each other, during unstructured or ‘free’ play.

## 2. Literature Review

### 2.1. Early Childhood STEM Integration and Play-Based Learning

STEM education in early childhood is primarily an interdisciplinary effort that integrates science, technology, engineering, and mathematics—often expanded to include the arts (STEAM)—to encourage creativity and critical thinking (Henriksen et al., 2019; Perignat & Katz-Buonincontro, 2019). Inquiry-based pedagogies (such as project-based and problem-based learning) offer experiential opportunities for young children to understand abstract concepts through activities that promote hands-on interaction and active participation (Herro & Quigley, 2017; Hossain et al., 2024; Quigley et al., 2017; Temiz & Çevik, 2024). Core elements of scientific inquiry, including engaging in mathematical modelling and engineering-design thinking, are integrated to provide both cognitive and physical preparation for future learning (Bertrand & Namukasa, 2020).

A substantial body of research supports free play as an enriching environment for STEM learning. Young children naturally explore basic engineering and mathematical concepts through play, especially in relation to construction, measurement, and the behaviour of materials (Gelir et al., 2024). Numerous observational studies have documented the frequent presence of rich “STEM talk” during cooperative and pretend play, as well as children’s engagement with physical phenomena, such as forces, magnets, and simple machines (Solis et al., 2017; Thibodeau-Nielsen et al., 2025). Adult intervention can enhance learning when it involves supported peer collaboration, fostering exploration through project and play-based activities (Gold & Elicker, 2020; Rushton & King, 2020). Playful experiences in maths and science are powerful foundations for children’s future academic success and have also been shown to be strong indicators of future learning outcomes (Cankaya et al., 2023; Clements & Sarama, 2016).

Although substantial evidence supports the use of play-based approaches as valuable environments for the early development of young children’s STEM knowledge, interpretations of how this form of play can be effectively facilitated by teachers or educators must consider the age-specific developmental characteristics of the children involved. To comprehend the development of STEM-related behaviours in early childhood students, it is essential to analyse their interaction with the environment and materials during play, as well as how their distinctive age-related ways of thinking and communicating influence the STEM behaviours demonstrated during these play experiences. From a sociocultural perspective, these types of play can be seen as mediated activities in which children develop their own STEM-related knowledge through social and material mediation, laying the groundwork for establishing early engineering habits of mind.

### 2.2. Developmental Characteristics of STEM Learning in 3–4-Year-Olds

Τhis section explores STEM learning in 3–4-year-olds from a pedagogical viewpoint, emphasising what is considered ‘playful’ and ‘socially interactive’ behaviour for their age.

Early childhood education is characterised by action-based exploration and social mediation of meaning, rather than abstract thinking or sustained conceptual reasoning (Evertsen et al., 2023). Young children (typically 3–4) tend to engage in STEM learning through hands-on manipulation of materials, repeated attempts, and trial-and-error processes (Clements et al., 2021). Children at this age tend to think very closely to their immediate experience, and their cognitive activity is generally expressed through movement, object manipulation, and responses to immediate feedback, rather than through long-term, verbally planned explanations (Bembich & Bologna, 2025). Therefore, behaviours typically described as planning, sequencing, or problem-solving in a pedagogical sense are developed through situational action (as opposed to planned strategies) (Piaget, 1977).

Furthermore, language at this stage is often used to accompany actions rather than precede them. Young children frequently use gestures, imitations, and joint attention to communicate their intentions or negotiate play, especially during collaborative play (Tomasello & Farrar, 1986). They also build understanding with their peers by observing, repeating, and responding to each other rather than through lengthy verbal exchanges (Heesen et al., 2017). These traits are visible in many aspects of STEM-based play.

STEM-based play between ages 3 and 4 can link children’s sensorimotor experiences with emerging symbolic representations (Ha et al., 2023). Repetition and imitation are key parts of young children’s play, allowing them to test hypotheses, find patterns, and predict results (Lockman & Tamis-LeMonda, 2021). In such repetitive and imitative play, STEM-related behaviours appear as exploratory actions, such as pressing buttons repeatedly, moving in specific directions, adjusting toy positions, and changing their movement responses based on observed outcomes (Ching & Hsu, 2024). These explorations reflect appropriate early forms of mathematical, technological, and engineering thinking (McCluskey et al., 2023).

From a pedagogical perspective, these features suggest that early STEM engagement should be seen through the child’s interactional processes (e.g., how they engage with others), rather than focusing solely on the outcome (e.g., whether they have mastered the concept) (Campbell et al., 2018). Recognising that planning, sequencing, and problem-solving at this age occur through socially situated, playful, and embodied activities offers a framework for interpreting children’s engagement with STEM ideas during free play in a developmentally appropriate way (Ha et al., 2023).

From a sociocultural perspective, these developmentally appropriate forms of play, being action-based and socially situated, will exemplify early signs of engineering habits of mind because children, through mediated interactions with materials and peers, develop ways to explore, iterate, and solve problems.

### 2.3. TETs as Tools for Early STEM Learning

Digital technologies, especially robotics, coding platforms, and other multimedia toys, are positively linked to children’s fine motor skills, language, and creative thinking; however, the quality and depth of children’s learning with TETs depend on the family and classroom environment (Hu et al., 2024; Bourha et al., 2024). TETs can be seen as cultural and mediational tools that help children engage in exploration, problem-solving, and early engineering habits of mind through social and material mediation. Additionally, the research on TETs indicates that using programmable robots, coding activities, and electronic blocks helps children understand engineering design reasoning, scientific inquiry about their surroundings, and computational thinking through playful, hands-on tinkering and experimentation (Adipat et al., 2021; Kewalramani et al., 2020; Kong, 2021; Lee et al., 2025; Murcia et al., 2020; Zhang et al., 2025).

The effectiveness of TETs also relies on contextual factors, such as the authenticity of the pedagogical approach. For example, when educators and teachers allow young children to explore devices like Bee-Bots or robotic animals during free play, without predefined instructions or tasks, these toys offer spontaneous learning aligned with children’s everyday experiences. Conversely, when the same toys are used in structured, pedagogically guided activities, engagement is often less sustained. Moreover, educators’ ability to meaningfully integrate TETs, along with access to professional development, is another key factor influencing their successful application in early childhood settings (S. Li, 2021; Madanipour & Cohrssen, 2020; Park et al., 2017; Pollarolo et al., 2024; Wan et al., 2021). Learning, collaboration, sequencing, spatial reasoning, and causality (cause-and-effect thinking) are supported when children are given opportunities to engage with open-ended materials and access digital tools and technologies (Bucher & Pindra, 2020; Sando et al., 2021). The literature is now more widely recognising the capabilities of TETs to assist children’s learning; however, there are barriers for educators when implementing TETs into their educational practices. These include limited digital confidence, a lack of pedagogical training in technology use, concerns about screen time, and challenges in promoting digital citizenship in developmentally appropriate ways (Marsh et al., 2018; Papadakis et al., 2021). Rather than focusing solely on the affordances of TETs, much current research emphasises the need to address these barriers through system-wide support. This underscores the need to develop sustainable, research-informed strategies that empower educators to implement TETs meaningfully, such as prioritising playful, child-led learning and aligning with the values and practices of early childhood education (Papadakis, 2021; Pollarolo et al., 2024).

Numerous empirical studies and meta-analyses provide strong evidence for the benefits of TETs in early childhood education contexts. Longitudinal and controlled interactions have identified enhancements to young children’s acquisition of scientific concepts, mathematical reasoning, spatial skills, problem-solving, creativity, and collaboration with the regular use of programmable toys and augmented reality TETs (Chen & Tippett, 2022; Hall et al., 2021; Nguyen-Viet et al., 2023; Pollarolo et al., 2024). Moreover, recent meta-analyses and longitudinal studies have demonstrated that when TETs are implemented using specific, developmentally appropriate pedagogical methods and educational curricula, they consistently yield positive results for children’s ability to learn across STEM domains and creative problem-solving (Burns et al., 2025; Lu & Xie, 2024; Luen et al., 2024). These benefits have the most significant impact when digital tools are incorporated into child-centred, collaborative, and exploratory environments where learning occurs through inquiry-based activities. While these findings are encouraging, they must be considered alongside the previous discussion of system-wide challenges, such as access inequities, the need for ongoing professional development, and concerns about age-appropriateness and screen time (Livingstone & Blum-Ross, 2020; Qin et al., 2021).

In conclusion, while TETs may provide young children with various early STEM learning opportunities, realising this potential requires intentional, context-sensitive, and systemic support for educators and children.

## 3. Materials and Methods

### 3.1. Research Design and Approach

This study employed a qualitative approach, using systematic observation and analysis, focusing on children’s interactions with technology-enhanced toys (TETs) during free play. Observational methods support early childhood research by capturing spontaneous, child-led behaviour and peer interactions in natural play settings (Edwards, 2017). Collecting data in this way facilitates an understanding of children’s learning experiences, and the nature of the play environment may either promote or hinder emergent behaviours in young children. Any numerical indicators presented (e.g., 35 references for Science with Bee-bot, 22 references for Engineering with Coko-robot) are employed descriptively to enhance qualitative interpretation rather than for statistical analysis.

The research questions guiding this study are:

1. What STEM-related skills and behaviours do children aged 3–4 demonstrate during free play with technology-enhanced toys in early childhood education settings?
2. How do different types of TETs support or restrict young children’s participation in STEM learning processes during free play?

### 3.2. Sample and Participants

The selection of ECEC settings was conducted using a purposive sampling strategy to ensure representation across diverse geographic and institutional contexts. Four ECECs participated in the study: one private centre located in a regional area outside the capital city of Athens and three centres in Athens (one private and two public ECECs). This sampling approach was designed to capture a diverse range of pedagogical practices, available resources, and contextual conditions within early childhood education settings.

The sample comprised 37 children (19 boys, 18 girls) aged 36 to 47 months. These participating children attended ECECs serving families from diverse socioeconomic and cultural backgrounds, in both urban and regional settings. Written informed consent was obtained from parents or legal guardians for all participating children.

It is important to note that while the participating ECECs served a variety of families from diverse socio-cultural backgrounds, the individual sociocultural attributes of both children and their families were not considered as analytical variables in this research project. The primary focus of the analysis was on children’s observable behaviours and the interactional processes that developed among children and TETs during free play. Rather than attributing observed behaviours to individual background factors, the study aimed to explore how children engaged in STEM-related activities through their interactions with peers and TETs within an educational setting.

To provide additional context for interpreting the study’s findings, the researchers documented descriptive data about each specific ECEC environment (e.g., type of service provider, rural or urban, available play space, and access to instructional materials and equipment) through field notes. These descriptive data provided context for the researchers’ interpretations of the play environments and the conditions under which these interactions occurred; however, they were not utilised to establish or compare subgroups of children. This approach aligns with the exploratory and pedagogical orientations of the study, which sought to examine children’s interactive processes in early childhood settings rather than to conduct comparative analyses based on children’s individual backgrounds.

### 3.3. Ethical Considerations

The research followed strict institutional and international guidelines for ethically sound educational research involving young children. Following a standard process, institutional ethical approval was obtained from the University of West Attica Institutional Review Board. Ethical review was required for all procedures, instruments, and consent forms to ensure compliance with national and international guidelines. Informed consent from the parental/guardian and age-appropriate assent from young children participating in the research were required.

The confidentiality of the data and the safety of the participants were a priority: all data were anonymised; all video/data files were encrypted and stored securely; all files were only accessible by authorised personnel; and the materials shared or published contained no identifiable information. The study also considered the General Data Protection Regulation (GDPR) in addition to compliance with national data protection laws. All procedures in the study included protocols for secure storage, controlled sharing, and data destruction after study completion.

Moreover, individual participation was voluntary, and children and their guardians could withdraw their consent at any time, with no repercussions for school attendance or the use of services that would continue years after the study ended. Researchers were experienced early childhood educators capable of responding sensitively to children’s needs, with procedures to minimise discomfort and risk. They used non-intrusive observations and age-appropriate technologies throughout the study. All research activities focused on the participants’ safety, dignity, and autonomy.

### 3.4. Technology-Enhanced Toys Used in the Study

As shown in Table 1, five technology-enhanced toys (TETs) were selected based on their developmental appropriateness and user-friendly design (Appendix A).

Each toy was introduced during free-play sessions, enabling children to explore and interact with the devices individually and collaboratively. The selection of these TETs ensured a balance between screen-free programmable robots and interactive toys with digital feedback, supporting a range of learning objectives and play experiences. Appendix A includes additional information about the photographs and the toys’ URLs.

### 3.5. Data Collection Methods

The methods used to gather data on children’s interactions with Technology Enhanced Toys (TETs) during free play relied on a qualitative approach that included video documentation and systematic written observations. Combining these two data collection methods provided a rich and authentic representation of children’s behaviour. In the regional ECEC, ten sessions were conducted over three months (November 2022–January 2023). Children in small groups (up to 5) were video recorded during their free play with TETs. Each session lasted approximately 30 min, totalling 257 min of video footage. Five additional sessions were video recorded at the private Athens ECEC (January to March 2023), producing 92 min of video data. Although the public ECECs in Athens did not permit video recording due to privacy concerns, methodological consistency was maintained across sites by taking systematic written notes during nine 30 min play sessions (May to July 2023), totalling 1757 min of data.

Systematic written field notes were taken across participating ECEC sites during each play session, recording detailed, context-rich information, including subtle bodily gestures, whispered verbal exchanges, group dynamics, and environmental factors that might influence play and interaction. The cumulative duration for all written observations was 1703 min, providing a rich qualitative dataset for future analysis. This approach enabled the capture of both initial learner behaviours and their subsequent changes over time, alongside spontaneous behaviours as they occur in situ (Colliver et al., 2022). Both video recordings and detailed observation notes enabled more thorough, richer data collection. A multimodal (video/observation) methodology provided a whole-child perspective on children’s experiences in their interactions with TETs, enabling the researcher to document overt and spontaneous, unplanned behaviours. The video material also allowed the researchers to review the areas of observation that were most complex or confusing at the time of observation and to validate their interpretations as they analysed the video. Observation field notes were expanded and triangulated with video documentation, broadening our understanding and narrative, and aiding in a thorough, context-based analysis of the entire data corpus.

In each free-play session, early childhood educators were present in the classroom to fulfil their usual roles as supervisors and to ensure a safe environment for the children. Educators did not participate in guiding activities, introducing tasks, or providing instructional guidance with TET use. The only times educators offered scaffolding support to help children’s play or directed children’s engagement with the toys involved safeguarding children from potential injuries or assisting in transitioning students through daily classroom routines. As a result, the observed play episodes showed that children initiated and directed their own peer-to-peer and TET interactions, rather than engaging in educator-led or pedagogy-based instructional approaches.

### 3.6. Data Analysis

Video and written data were aligned, cross-referenced, and systematically integrated throughout the analysis process. All qualitative data, including transcriptions of video recordings and field notes, were imported into NVivo 15 (version 15.3.2), a qualitative data analysis software (CAQDAS). Initial coding was conducted inductively, with codes developed directly from observed patterns of behaviour during free-play sessions. Video transcriptions were segmented and coded according to interactions between children and TETs. The emergent themes included children’s ability to manipulate technological features, verbal and nonverbal interactions, collaborative efforts, and problem-solving methods.

These initial inductive codes were then examined to identify recurring patterns of actions, communicative behaviours, and interactional responses related to children’s engagement with TETs during free play. This involved an iterative process of reviewing, comparing, and refining patterns across clustered codes to form broader thematic categories. Similarities and differences among the patterns were scrutinised, overlaps resolved, and conceptual clusters developed within thematic groupings. Observed behaviours were interpreted as STEM-related based on their alignment with one of the previously identified early childhood STEM indicator types, namely sequencing, prediction, iteration, problem solving, spatial reasoning, or cause-and-effect reasoning, as derived from earlier research on early childhood STEM engagement. The study did not interpret these indicators as “competencies” but rather as behaviours emerging from children’s interactions with peers, materials, and the toys’ technological features during play. This approach enabled the study to identify STEM-related themes grounded in observable practices within its qualitative and interpretive framework.

Additionally, field notes were connected to video excerpts from specific sessions. These written notes provided context for what occurred prior to and during the session, including prompts and reactions from peers, as well as environmental conditions that affected the child’s ability to interact effectively with TETs (e.g., crowded play space that limited movement, technical malfunctioning in the TET, or interruption from other children). The extensive field notes enhanced the overall understanding of the children’s actions and strengthened the triangulation of evidence. Data entry into NVivo 14 enabled researchers to cross-reference datasets (e.g., comparing children’s verbal explanations from video with related field notes or coding references) to identify consistent themes and commonalities across datasets. For example, children who frequently used the Bee-bot’s directional commands could be identified through multiple coding categories, including exploratory play and emergent mathematical thinking, allowing researchers to identify overlap across datasets. This organised, iterative approach to analysis increased analytical rigour and yielded reliable results through systematic comparisons, visual representations of coded data, and the ongoing development and refinement of each theme. The counts of coded references are presented solely as descriptive markers to illustrate engagement patterns within toys and STEM areas, without suggesting any quantitative measurement, comparison, or statistical inference.

A researcher with expertise in early childhood education and qualitative observational methods conducted all coding and initial analysis for this study. Iterative and reflexive coding techniques were used to improve both the rigour of the analysis and their own reflexivity. This involved repeatedly reviewing the video data along with emerging codes and definitions across multiple analytic cycles. Peer debriefing and supervisory discussions occurred throughout the analysis to evaluate coding decisions, refine code definitions, and critically interpret the data. As this qualitative study focused on meaning-making and context-specific interpretation, formal inter-rater reliability statistics could not be used to measure coder agreement. Instead, the validity of the findings was supported through prolonged engagement with the data, triangulation of different data types (video and written observations), and ongoing iterative review. Table 2 summarises STEM-related indicators and provides representative examples of behaviours that may suggest their presence.

## 4. Results

### 4.1. Overview of STEM-Related Behaviours in Free Play

By systematically observing young children engaging with TETs during free play, meaningful aspects of STEM-based behaviours, such as curiosity, exploration, problem-solving, reasoning, and collaboration, were identified for the specific age group (3–4 years old) across all four settings. The duration and quality of children’s involvement with STEM activities varied considerably, even though they were all approximately the same age. Children exhibited varying levels of engagement with TETs: some vocalised their intentions before attempting to solve the problems, while others remained silent, persistently exploring and seeking solutions. Consequently, the findings are presented as ranges and developmental patterns typical of the age group, rather than as uniform skills or capabilities in individual children.

These observations demonstrated how young children develop essential STEM skills in different ways, influenced by their age, environment, and resources. Results are organised into the four STEM areas.

### 4.2. Science: Observation, Exploration, and Inquiry

When used during child-led play, TETs frequently prompted curiosity and scientific thinking. Across 78 coded references (8.44% of total observations), children demonstrated a range of behaviours associated with fundamental scientific inquiry, including manipulating objects, exploring the capabilities of toys, and developing informal hypotheses. For example, children made predictions when interacting with toys based on previous experiences with similar toys (“D. uses his hand to tap head on the dog to start the action, believing it is similar to the toy he has at home”) and modified play based on problem-solving when children were interacting with Coko-robot and its path to avoid obstacles and furniture. These behaviours occurred predominantly in contexts with open-ended, interactive toys, play areas that offered flexible opportunities for exploration, and peer interactions that supported collaborative inquiry. Consequently, the characteristics of the learning environment and available resources influenced the emergence and complexity of children’s scientific behaviours (Table 2).

### 4.3. Technology and Engineering: Problem-Solving and Tool Use

Many references (N = 492) were coded under the category of children’s engagement with technology and engineering as they interacted with TETs, often highlighting the secondary affordances of the devices. For example, while the Bee-bot was primarily designed to support sequencing and programming, children also engaged with its other design elements, such as its sounds, movements, and button responses, which provided opportunities to explore problem-solving strategies. These additional design features enabled children to investigate the device’s reaction to different inputs, correct navigation errors, and test hypotheses with their peers. Such iterative actions mirror some fundamental engineering behaviours, such as debugging, adjusting space, and modifying a plan based on feedback. During free play, children explored the affordances of the devices and solved mechanical problems through creative experimentation. For example, Maximus (age 3.6 years) comments, “I pressed ‘OK’ on the Coko Robot, I put it in the box, and then I saw what happened.” Children also showed familiarity with basic operational features of TETs, such as changing laptop batteries or entering and testing command sequences on the Bee-Bot (Table 2).

Children appeared to develop computational thinking tasks with the Bee-bot and the Coko Robot. They also predicted and sequenced commands for Bee-bot, verbalising their thinking, e.g., Philip (age 3.5 years) notices: “If I press this twice, it turns! But wait…I want to go back!” Children demonstrated logical thinking and iterative mistakes. Engineering practices in free play emerged when children experimented with assembling devices, modifying features, and testing to achieve design goals, such as altering the Coko-Robot’s path trajectory or avoiding barriers, e.g., changing the direction the Coko was facing after running into furniture. These actions demonstrated the design cycle and iterative learning process, where children planned, tested, and revised their actions to solve a challenge (e.g., placing different tiles on the Coko-robot and observing the resulting outcomes).

There were also examples of independent problem-solving, as children took action to troubleshoot without needing adult assistance. They made decisions independently, such as activating an operation function, resolving technical issues, inserting new batteries, or adjusting the Bee-bot inputs, based on the feedback they received. These examples collectively identify that children developed emerging engineering and technology skills in a non-prescriptive way through free play with TETs.

### 4.4. Mathematics: Patterns, Counting, and Spatial Awareness

The engagement of children with TETs contributed to the development of counting, spatial orientation, and the beginning of patterning and repetition, key components of early mathematical thinking (93 references, 10.06%). Evidence of using patterns featured prominently when children repeated numerical or colour orders to activate toy responses or anticipated that repeating the same action would yield the same predetermined outcome.

For instance, children such as K. and G. (3 years old) demonstrated sequencing when pressing buttons in an ordered manner (e.g., “K. said: ‘I pressed all the buttons!! 1, 2, 3, 4!’”), demonstrating their emerging understanding of order and repetition (Table 2). Repetition was also noticeable when children caused or anticipated the same outcome, such as pressing the buttons on the Owl and the Laptop to repeatedly produce a well-known song cue. This was an early example of pattern recognition and anticipation. In another example, when using programmable toys such as the Bee-bot, children typically programmed movement paths by repeating directional commands (e.g., “forward, left, forward, left”), creating and following a simple movement pattern. These repetitive actions indicated regularity and anticipation in a play context.

These observations suggest that early indications of patterning, as observed in children’s numerical sequencing, repeated-sound responding, and directional programming, highlight their engagement with early mathematics, allowing the emergence of regularity, repetition, and predictive reasoning in play-based learning experiences.

Summarising, Table 2 provides an overview of all STEM-related components identified in children’s free play with Technology-Enhanced Toys (TETs), including relevant behavioural indicators for each of the four STEM domains, brief descriptions of each indicator, and supporting evidence from the observational data.

### 4.5. Differences Across the Types of TETs

Upon analysing 654 coded segments, children exhibited STEM-related behaviours according to the opportunities presented by TETs. However, the specific types of skill-set behaviours tended to align with the affordances and open-endedness of each TET, rather than with the toy itself.

When engaging with programmable and open-ended toys, children aged 3–4 began to exhibit behaviours related to STEM as they developed their own ways of thinking about STEM concepts. Many children demonstrated some degree of planning and directional thinking while playing with the Bee-Bot, experimenting with different command sequences and observing how the Bee-Bot responded to them. Although children varied in their ability to articulate what they were doing verbally, some children were observed trying to communicate their intentions (e.g., “Go There!” or “Turn!”) and making attempts to adjust their input based on the toy’s response. Some of these interactions were very short. However, they indicated that children initially understood cause and effect, spatial reasoning, and problem solving through trial and error. It was also noted that during shared play with the Bee-Bot, some children engaged in collaborative interactions by pointing, commenting, or imitating one another’s actions. The open-ended, tactile design of the toy seemed to foster curiosity, creativity, and shared focus, suggesting its ability to help children develop fundamental STEM engagement skills throughout early childhood.

When engaging with the Coko-robot, evidence of engineering habits of mind, such as cause-and-effect reasoning and iteration, was also apparent. Children planned and re-adjusted their strategies when the toy’s movements were impeded, verbalising their purpose and adapting to feedback provided by their surroundings (e.g., “We need to put the object in the box and press the OK button. Maybe it will go now”). These episodes involved children engaging in experimental thinking and conducting initial design cycles as they planned and conducted experiments, observed the results, and adjusted their actions to achieve their intended outcomes.

Children participated in basic numeracy, imitation, and symbolic play using less engaging, non-interactive toys, such as the Owl and the Laptop. For example, engagement with these toys often involved much more repetition (e.g., “One, two, three! It sings again!”) and less opportunity for extended experimentation or problem-solving. While these are useful and help build foundational skills, such conditions are less likely to foster exploratory STEM behaviours or collaborative troubleshooting.

Overall, the data provided evidence that children’s development and demonstration of STEM skills were highly aligned with the opportunities for flexible play offered by the TETs. Open-ended toys, such as the Bee-Bot and the Coko-robot, encourage collaborative working, critical thinking, and sustained inquiry behaviours. In contrast, closed-ended toys with predetermined responses reinforce basic, often repetitive skills. Early STEM engagement and the extent to which a child experiences it are better understood through how their behaviour during exploration, negotiation, and interaction with the toy reflects the quality of that experience, rather than just the level of technology in the toy itself (see Table 3).

The following table shows the total number of observational reference points coded for each TET across the four STEM domains. It shows that different toys prompted different STEM-related behaviours at varying frequencies during free play.

Overall, the results indicate that technology-enhanced toys (TETs) support children’s STEM-related skill development, particularly when they feature programmable capabilities and offer opportunities for free play. Such toys enable broad participation across STEM domains, whereas toys with limited interactivity provide fewer opportunities for cognitive development. The nuances of holistic STEM learning and disciplinary skill development will be discussed further in the discussion section.

## 5. Discussion

This study investigated the STEM-related behaviours of 3- to 4-year-old children during free play with TETs in four early childhood education and care settings in Greece. While most international STEM literature examines STEM behaviours with children aged five and above (Burns et al., 2025; Clements & Sarama, 2016), this research provides empirical insight from a younger cohort.

Using both video recordings and field observations, the study captured spontaneous, real-time interactions between children and TETs. It is essential to distinguish observed play conditions in this study from their pedagogical implications. The results from child-initiated free play are based on an absence of deliberate educational intervention by educators. Consequently, references to educator support for early STEM development reflect both interpretations of the observed interactions and the theoretical implications derived from them. Mentioning educators’ potential to facilitate play-based STEM engagement suggests ways they might enhance or encourage similar interactions with students, while clarifying the non-directed nature of the pedagogical context in which the data were collected. By documenting the emergence of STEM-related behaviours in children’s free play, this naturalistic approach provided insights into children’s developing problem-solving abilities, experimentation, collaboration, and early manifestations of computational thinking.

The significant contribution of this research lies in its methodological stance. Most existing research on children’s STEM knowledge has predominantly focused on older children, typically ages five to twelve (Wan et al., 2021), and has relied on surveys, teacher reports, or structured tasks that assume children will provide verbal fluency, metacognitive awareness, and adult-led engagement. Younger children, on the other hand, demonstrate their understanding through actions, gestures, and spontaneous exploratory behaviour rather than through articulate verbal explanations.

Using systematic video and written observational methods, this study produced rich, detailed data that demonstrated how young children interacted with TETs in natural and child-initiated settings. This method of data collection identified subtle, socially embedded behaviours, such as collaborative problem-solving, iterative experimentation, and deliberate interaction with digital and robotic toys, which more traditional, adult-directed data-collection methods may miss. As such, this study added new knowledge about how fundamental STEM-related behaviours are developed before the age of five and highlighted the need for additional research conducted in a more naturalistic and developmentally appropriate manner to inform early STEM education.

Findings indicate that children as young as 3 years old engaged in behaviours indicative of early STEM-related thinking (RQ1), including computational thinking, sequential reasoning, problem-solving, and collaboration. The clearest examples of these behaviours were observed during interactions with open-ended, programmable toys, such as Bee-Bot and Coko-Robot. During these play episodes, children verbally issued commands, predicted movement patterns, tested different solutions, and corrected errors. These actions demonstrated an emerging ability for logical reasoning and iterative thinking (Misirli & Komis, 2023; Pellas, 2025). For instance, as shown in Table 3, playing with Bee-Bot generated 35 science-coded references, 28 technology-coded references, and 25 engineering-coded references.

Children’s actions were primarily driven by their interactions with TETs. While the toy’s technological capabilities can stimulate children’s imagination and provide a novel play environment, the nature and quality of children’s interaction with the toy are equally, if not more, critical determinants of the play experience. Children’s verbal reasoning, hypothesis testing, and problem-solving skills were stronger when the toys allowed flexible, trial-and-error play and facilitated peer interactions. Thus, these findings support the notion that STEM engagement occurs through social participation and exploratory behaviour, rather than focusing exclusively on technological affordances.

Children engaged in peer negotiation of ideas, observed cause-and-effect, and shared their discoveries with others while using the toys. These findings support prior research indicating that young children can engage meaningfully in STEM learning through self-directed exploration (Thibodeau-Nielsen et al., 2025). In the observed episodes, TETs served as mediational tools through which children engaged in scientific inquiry, collaborative learning, and the social construction of understanding during play.

A crucial finding of this study was that young children engaged differently with various types of TEΤs, with these differences emerging through observable patterns of interaction during free play. In the present data, open-ended programmable toys (e.g., Bee-Bot, Coko-Robot) were associated with longer engagement, more repeated experimentation, and longer problem-solving sequences than closed-ended toys (e.g., Owl and Laptop) or toys with specific/limited functions (e.g., Dog Robot). For instance, the Owl and the Laptop primarily elicited elementary symbolic and numerical responses and rarely led to exploratory or iterative STEM-related activity (Table 3). These patterns align with the findings of Komis et al. (2021) and Murcia et al. (2020), which assert that the openness and flexibility of toy design are important factors in affording richer opportunities for STEM engagement. Importantly, it is not simply the toy itself; it is how the toy enables children to act, experiment, and negotiate meaning within self-directed play. This aligns with Edwards (2017), who argues that when digital play is effectively designed to fit into children’s everyday lives, it introduces new ways for children to socially, physically, and cognitively explore their world. In this research, very young children demonstrated extended and multidimensional STEM behaviours when toys allowed them modify actions, test outcomes, and collaborate with peers, illustrating the importance of designing both technological tools and play contexts to support mediated, exploratory interaction.

The toys utilised in this research represented more than just learning tools. They served as mediational devices, allowing young children to develop knowledge based on their actions and social interactions. From a sociocultural perspective (Vygotsky, 1978; Fleer et al., 2014), these interactions can be understood as early expressions of engineering habits of mind, such as iterative testing, problem-solving, and collaborative reasoning, enacted through children’s engagement with technological materials during free play. This research adds to an increasing body of literature showing that free play and STEM education are not mutually exclusive but instead work well together as different forms of instruction (Arnott, 2018; Edwards, 2017). Child-led free play was used as an observational pedagogical condition within the research design; children had the freedom to use TETs at their own pace, make their own choices about whether to engage or disengage, and operate the technology independently without adult guidance. The study’s results indicate that there are possible ways to support child-led free play through developmentally appropriate teaching methods to help children explore STEM subjects (Zeng & Ng, 2025). From a pedagogical standpoint, for young children, guided-discovery is not necessarily instructional in nature; it can include very minimal and contingent supports (e.g., asking a question at the right moment, encouraging children to reflect on their work, providing additional materials based on children’s emerging interests) that allow teachers to provide scaffolding while still allowing children to have control over their own learning. These pedagogical aspects are presented as conceptual implications and were not examined empirically in the study.

TETs embedded within richly contextualised play environments may function as “possibility tools” (Bourha et al., 2024) and provide developmentally appropriate entry points for young learners to begin understanding central STEM concepts while promoting exploration, agency, and experimentation.

The observed patterns in the study align with broader trends reported in recent meta-analyses and systematic reviews, which have highlighted the potential of early STEM experiences to support problem-solving and collaboration (Burns et al., 2025; Çelik et al., 2025). In alignment with previous studies suggesting that children naturally use STEM-related terminology and conceptual knowledge in their imaginative and peer-led play (Thibodeau-Nielsen et al., 2025), this study also concluded that children aged 3–4 spontaneously verbalised ideas related to sequencing, measurement, directionality, and logical reasoning during free play with TETs. However, the current study builds upon previous work by specifically highlighting the importance of open-ended toy design, which offers ample opportunities for creativity, imagination, and multimodality.

Previous studies have identified an ongoing need for additional professional development for pre-service and in-service early childhood educators regarding their digital competence and ability to utilise STEM-related technology (Dardanou et al., 2023; Papavlasopoulou et al., 2024). They have also highlighted an ongoing lack of institutional support in this area (Papavlasopoulou et al., 2024). For even the most effective TETs to be fully utilised, they must first feel confident and capable of utilising these technologies meaningfully (Amemasor et al., 2025; Boz, 2023). Therefore, it is evident that the issue of providing equitable opportunities for all children to learn STEM during the early years of education is not just about having access to the necessary toys/tools, but rather about supporting educators’ development with a strength-based approach to increase STEM engagement (Fleer et al., 2014), educational institutions’ structure, and providing policymakers the appropriate level of attention to this area.

Internationally and in Greece, policies for early childhood education have shifted toward an emphasis on inquiry-based, integrated STEM learning (Hurst et al., 2019). Policies that reflect this direction emphasise the value of encouraging children to explore and solve problems in interdisciplinary ways, rather than being restricted by rigidly defined disciplines. This research supports the paradigm shift by demonstrating that even very young children can begin learning about STEM through their play interactions when using technology-enhanced toys (TETs).

This study aimed to deepen the understanding of the role TETs play in early childhood education by observing how children utilise these toys during independent free play and how they support early STEM learning. The researchers employed observational methods to document various ways children interacted with TETs during self-directed play, thereby capturing behaviours indicative of early STEM learning, such as problem-solving, spatial reasoning, foundational mathematics and science thinking, and emerging computer science skills. Although the findings did not provide conclusive evidence of children’s STEM development, they underscored the pedagogical value of offering children opportunities for hands-on exploration of age-appropriate technologies to promote early STEM learning. As a result, this form of interactive play can serve as a foundation for fostering interests in STEM-related concepts as children progress through their educational journeys.

Overall, the study’s findings should be considered as exploratory and illustrative, showing how STEM-related behaviours may emerge through play-based interactions with TETs, rather than as evidence of consistent or causal effects.

## 6. Limitations and Suggestions

This study examined STEM-related behaviours in children aged 3 to 4 years across four early childhood settings in Greece, using written and video observations. Methodologically, the unequal distribution of participants across the four ECECs may have affected the density of behaviours observed in each setting and their variability, thereby introducing a bias toward stronger behavioural patterns. Furthermore, although the qualitative, naturalistic methodology used here is well-suited to capturing the nuances and contextual aspects of young children’s digital play, the heavy reliance on researcher interpretation during both coding and analysis reduces the likelihood of replicating the findings.

Another limitation of the present exploratory study was that, although the children participating in the study attended Early Childhood Education and Care (ECEC) programmes representing diverse socio-economic and cultural settings, their socio-cultural backgrounds were not used as analytical variables. As a result, the study did not explore how socio-cultural factors might influence variations in children’s access to materials, their interaction styles with these materials, or their engagement with TETs. The study’s naturalistic, play-based approach provided valuable data on how children interacted across various ECEC environments; however, future research could employ comparative or mixed-methods designs to examine children’s interactions with technology-enhanced toys across different socio-cultural contexts, incorporating insights from parents or educators to supplement the collected data.

Although the total sample size (N = 37) was large enough to provide an adequate amount of rich and contextually accurate insight into STEM learning in children less than four years old (a population of children who are infrequently studied directly via observation), additional research will be needed to create a more complete understanding of this complex developmental phenomenon. A larger sample size with greater demographic diversity, multiple-site comparative analysis, and mixed-methods research approaches, including quantitative observational data, educator interviews, artefact analysis, and other data collection methods, are examples of research opportunities to advance knowledge about young children’s use of technology-enhanced play. Longitudinal and experimental research designs could also potentially be used in future studies to assess the developmental progression of young children’s STEM learning and the causal effect of specific technology-enhanced play experiences on that development.

## 7. Conclusions

This study offers qualitative, observational insights into how TETs are utilised during free play, highlighting their role in fostering early engagement with STEM concepts among very young children. Data demonstrated that open-ended programmable TETs support various foundational STEM skills, underscoring the need for innovation, interaction, and pedagogical considerations in the design of future TETs.

This work identifies several key areas that require further investigation. The role of teachers in providing supportive learning contexts in STEM research in early childhood, in the design of TETs, and in selecting, using, and integrating technology-enhanced play to support children’s STEM skills. Additionally, equity issues should be considered: research and policy attention are required to ensure that all young learners have equitable access to high-quality TETs and that early childhood teachers have access to tailored, ongoing professional support. (Rad et al., 2022).

In conclusion, digital play has evolved over the years. Recent technological advancements in artificial intelligence will once again reshape how toys are designed and used. Play, in whatever form it takes, remains an invaluable and essential opportunity for early childhood development, allowing children to explore, experiment, and learn in meaningful ways. When integrated thoughtfully, technology-enhanced play can foster curiosity, collaboration, and early STEM learning. Therefore, whether it is physical, digital, traditional, or technologically mediated, play will always be the primary and non-negotiable mechanism through which young children develop, grow, and make sense of their world.

## Figures and Tables

**Table 1:** Selected TETs.

Toy Name	Description	Key Features	Educational Focus
Fisher Price Owl & Laptop	An interactive set featuring a talking owl and a child-friendly laptop.	Light-up buttons, music, simple games, voice cues	Early literacy, numeracy, and fine motor
Bee-Bot	A programmable floor robot shaped like a bee.	Directional buttons, memory, movement, and lights	Sequencing, computational thinking, logic
Coko Robot	A modular, programmable robot that can be assembled and coded by children.	Snap-together parts, coding cards, and movement	Problem-solving, coding, and collaboration
Dog Robot with Remote Control	Remote-controlled robotic dog with interactive responses.	Remote control, sound effects, movement, sensors	Social interaction, cause-and-effect, and empathy

**Table 2:** Explanation of the results of STEM elements with TETs.

Element	Indicator	Description	Examples
Science	Curiosity and exploration	Children exhibit curiosity about the mechanics of their environment through enquiry, observation, and manipulation of materials.	1. “D. strikes the dog’s head with his palm to activate it, since he assumes it will function similarly to a toy he had at home.” 2. “When Coko encounters an impediment, children alter its trajectory, and they likewise adjust it upon colliding with furniture.”
Technology	Problem-solving and interactive engagement	Children use technology to evaluate ideas, address obstacles, and accomplish tasks, demonstrating early computational thinking and logical reasoning. Children engage in practical, hands-on activities with technology, including coding robots, using touchscreen tablets, and playing digital games, which encourage experimentation and teamwork.	“M. engages with the Coko Robot, then gets on it and observes its responses. Subsequently, he places the toy in a box and presses OK, anticipating its response. Subsequently, the Coko robot was positioned on the floor and returned to the box, pressing OK once more to observe the mode of operation and movement”.
Engineering	Experimental use of TETs	Children experiment with TETs’ properties to effectively use them and achieve the desired results.	O. puts the batteries in the laptop.
Maths	Early math skills	Children count button presses and use directional language and gestures when programming TETs.	George wiggles to the owl’s songs, counts when he hears the numbers, and sings the songs he hears.

**Table 3:** Toy-related references across various STEM fields.

TET	Science	Technology	Engineering	Mathematics
Bee-bot	35	28	25	0
Coko-robot	12	30	22	14
Owl & Laptop	6	10	5	12
Dog-robot	2	4	3	1

Note: Video and written observation field notes were coded using NVivo. Each reference is a qualitative interpretation of STEM indicator behaviour (e.g., sequencing, prediction, experimentation). Descriptive and illustrative frequencies assist qualitative understanding rather than magnitude, prevalence, or statistical comparison.

**Figure A1:**
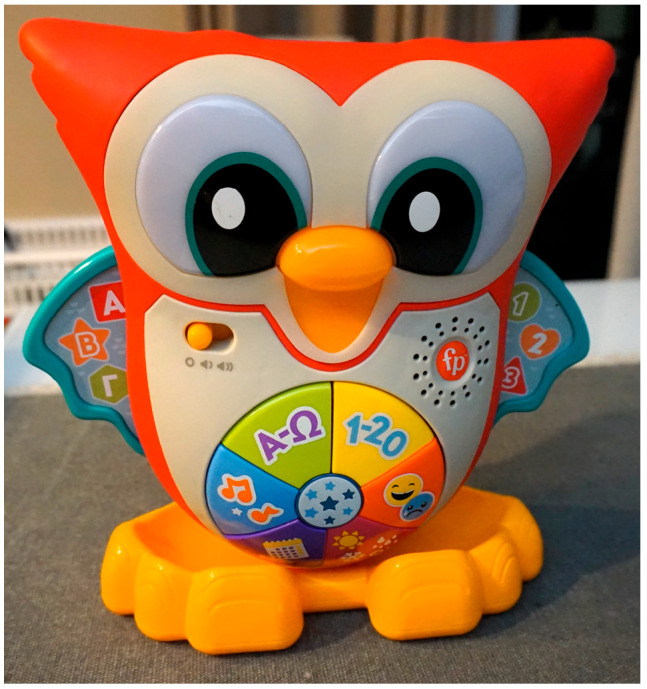
Amazon.com: Fisher-Price Toddler Learning Toy Linkimals Light-Up & Learn Owl for Ages 18+ Months, Compatible Only with Linkimals Items: Toys & Games (https://www.amazon.com/Fisher-Price-Linkimals-Light-Up-Interactive-Learning/dp/B09NP97B3Q#:~:text=Toddlers%20will%20abso-hoot-ly%20adore%20the%20Linkimals%20Light-Up%20%26,to%20activate%20lights%2C%20sounds%2C%20learning%20songs%20and%20phrases (accessed on 14 May 2025)).

**Figure A2:**
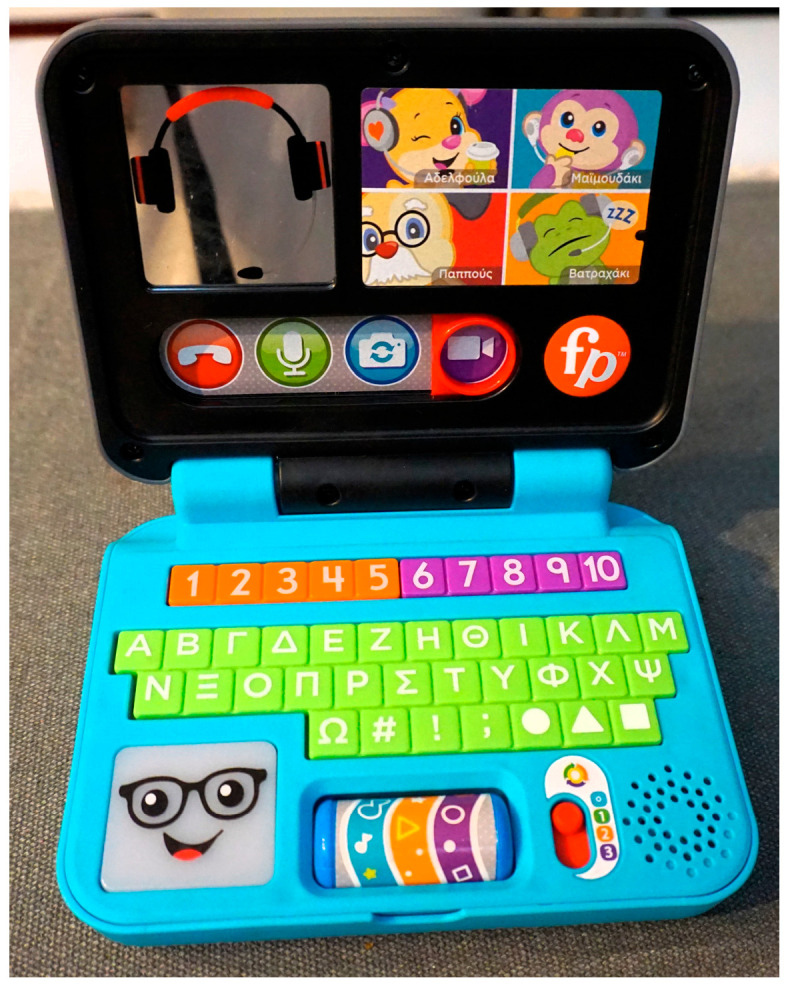
Amazon.com: Fisher-Price Baby & Toddler Toy Laugh & Learn Let’s Connect Laptop Pretend Computer with Smart Stages for Infants Ages 6+ Months: Toys & Games (https://www.amazon.com.au/dp/B09BDBKXFQ?ref_=mr_direct_us_au_au&showmri (accessed on 8 November 2025)).

**Figure A3:**
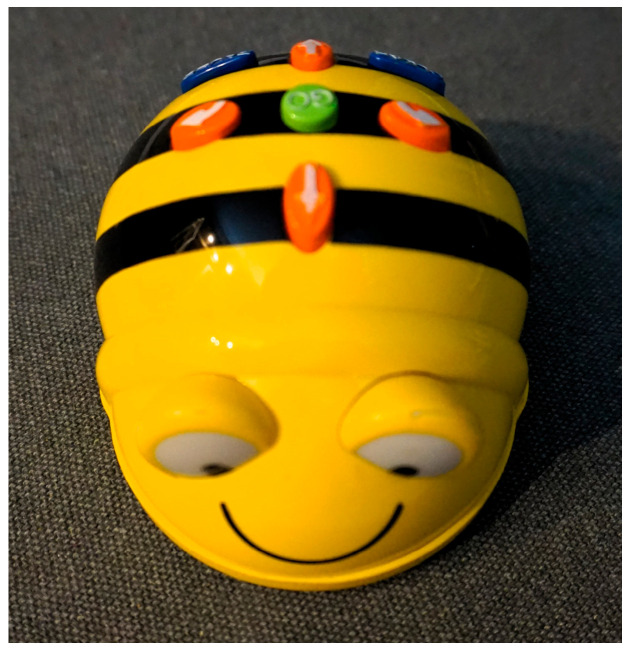
Amazon.com: TTS Bee-Bot Programmable Floor Robot: Industrial & Scientific (https://www.amazon.com/TTS-Bee-Bot-Programmable-Floor-Robot/dp/B08BTJFYT3/ref=sr_1_35?dib=eyJ2IjoiMSJ9.klcKXqlAEJR4Js37Veau9ahjTBaLCiNjosoTIQd0VOjHtqPv7EgOJEXAwebJLDE8ZL3Jnom-0fdj-oRzl5Q0WqP6sfn3jWHBHFCitvbX8hHbnaJUeKLIr-FYWc_2RW83uDhp1jdKKVTUd9hFF0Yae42NFGkTFEucy4auOM_B9iZf6VNOppIQSvxDkzBytchizkFvWGoNmk_umge2dYKH6x3tbSMSdBrCIKMxdRwNsVCp1ZzrqAvndRWDUfjzV5aNYCLF9WWjAqaAj02-dro2rdtSNEk0waCmNy6WmvJoG5w.yF0s6ZCVDb9ssYvhdgH-wXoK7UWI5FmLT9_18r4Wu9M&dib_tag=se&keywords=Bee-Bot&qid=1768941944&sr=8-35 (accessed on 14 May 2025)).

**Figure A4:**
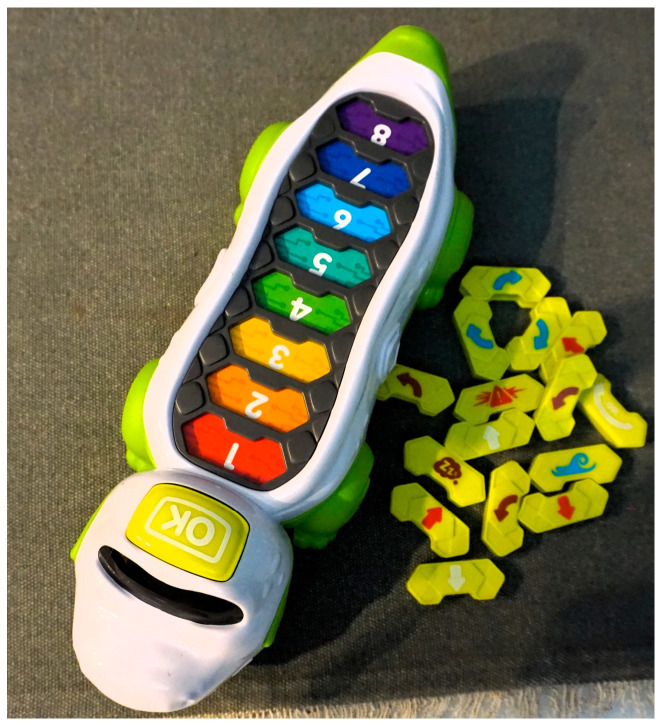
Clementoni-52384-Coding Lab-Coko, Programmable Crocodile Robot: Amazon.fr: Toys & Games (https://www.amazon.fr/-/en/Clementoni-52384-Coding-Programmable-Crocodile/dp/B07N6S3NT6 (accessed on 7 September 2025)).

**Figure A5:**
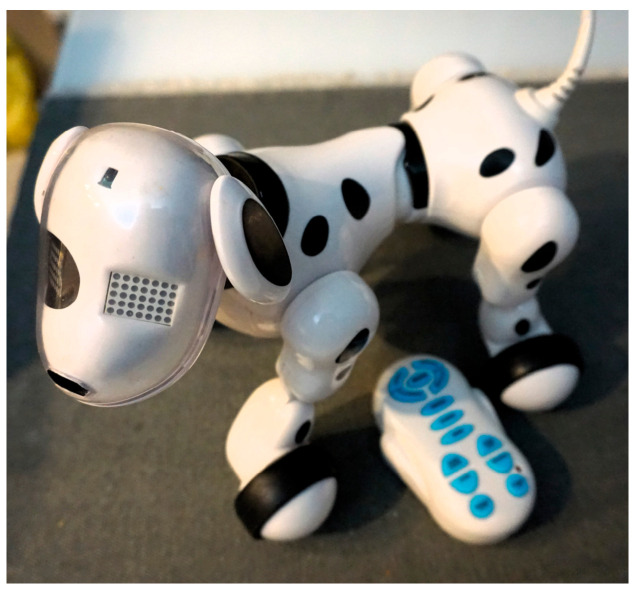
Lexibook, Power Puppy, My Robot Dog Learned to Trainer, Programmable Robot with Remote Control, Training and Gesture Control, Dance, Music, Luminous Effects, Rechargeable, DOG01LXB: Amazon.fr: Toys & Games (https://www.amazon.fr/Lexibook-Programmable-Training-Rechargeable-DOG01LXB/dp/B0FVXW54ZY/ref=sxbs_pa_sp_search_thematic_btf_sspa?content-id=amzn1.sym.c278443b-0330-4b3c-84a2-c9f3ceb0f564%3Aamzn1.sym.c278443b-0330-4b3c-84a2-c9f3ceb0f564&crid=24UND8E17XZ3&cv_ct_cx=robot%2Bpour%2Bchien&keywords=dog%2Brobot&pd_rd_i=B0FVXW54ZY&pd_rd_r=dc34d487-0895-4f47-846e-4a7fc1165e2d&pd_rd_w=hkG0N&pd_rd_wg=c7Mml&pf_rd_p=c278443b-0330-4b3c-84a2-c9f3ceb0f564&pf_rd_r=W3HFSZBYM8YCFJPXPVNN&qid=1768942880&sbo=RZvfv%2F%2FHxDF%2BO5021pAnSA%3D%3D&sprefix=dog%2Brobot%2Caps%2C315&sr=1-97-61680d47-1983-460a-81e0-80c8eefa0274-spons&xpid=RivHQQnV4t_t_&aref=DqUIiz9elc&sp_csd=d2lkZ2V0TmFtZT1zcF9zZWFyY2hfdGhlbWF0aWNfYnRm&th=1 (accessed on 14 May 2025)).

## Data Availability

The original contributions presented in this study are included in the article. Further inquiries can be directed to the corresponding author.

## Abbreviations

The following abbreviations are used in this manuscript:

STEM	Science, Technology, Engineering, Mathematics
TETs	Technology-Enhanced Toys
ECEC	Early Childhood Education Center
